# Occurrence, Distribution and Risk Assessment of Biocides in Chao Lake and Its Tributaries

**DOI:** 10.3390/toxics13111001

**Published:** 2025-11-20

**Authors:** Longxiao Ji, Lei Jiang, Shengxing Wang, Xiaozhen Hu, Kaining Chen, Qinglong Wu, Lijun Zhou

**Affiliations:** 1 School of Environmental Science and Engineering, Nanjing University of Information Science and Technology, No. 219, Ningliu Road, Nanjing 210044, China; jilongxiao@niglas.ac.cn (L.J.); zdhuxiaozhen@163.com (X.H.); 2 State Key Laboratory of Lake and Watershed Science for Water Security, Nanjing Institute of Geography and Limnology, Chinese Academy of Sciences, Nanjing 211135, China; jianglei212@mails.ucas.ac.cn (L.J.); 19544556822@163.com (S.W.); knchen@niglas.ac.cn (K.C.); qlwu@niglas.ac.cn (Q.W.); 3 University of Chinese Academy of Sciences, Beijing 100049, China; 4 School of Life Sciences, Institute of Life Science and Green Development, Hebei University, Baoding 071002, China; 5 Collaborative Innovation Center of Technology and Material of Water Treatment, Suzhou University of Science and Technology, Suzhou 215000, China; 6 Sino-Danish Center for Science and Education, University of Chinese Academy of Sciences, Beijing 100039, China; 7 The Fuxianhu Station of Plateau Deep Lake Research, Chinese Academy of Sciences, Yuxi 653100, China; 8 Center for Evolution and Conservation Biology, Southern Marine Sciences and Engineering Guangdong Laboratory (Guangzhou), Guangzhou 511458, China

**Keywords:** Chao Lake, biocides, occurrence, ecological risk, human health risk

## Abstract

Biocides, including fungicides and paraben preservatives, are widely used in medicine, agriculture and food industries, and are ubiquitous in aquatic environments, which will have adverse impacts on aquatic organisms. This study investigated the occurrence, distribution, ecological risks, and human health risks of 7 target biocides in Chao Lake, a large eutrophic urban lake, and its tributaries. Four biocides were detected, with total concentrations ranging from 186 ng/L to 853 ng/L. Carbendazim (CBD), fluconazole (FCZ), and methylparaben (MP) had detection frequencies of 100%, with mean concentrations of 234 ng/L, 35.3 ng/L, and 26.8 ng/L, respectively. Significant spatial heterogeneity was observed, with obviously elevated levels in the western region compared with the central and eastern regions. Strong correlations (*p* ≤ 0.01) were found between these three biocides, CBD, FCZ, and climbazole (CLI), and the following two environmental factors: total nitrogen and dissolved total nitrogen. Based on the risk quotient (RQ) evaluation, CBD was identified as a high-risk compound for aquatic organisms, particularly *Daphnia magna*, with RQ values exceeding 1 and reaching up to 7.42. CLI showed moderate risks at some sampling sites, while FCZ and MP posed no risk. Human health risk quotient (RQ_h_) analysis revealed no significant health risks to different age groups, with the RQ_h_ values of biocides at all sampling sites in Chao Lake below 0.1. The ecological risks of CBD warrant even greater attention.

## 1. Introduction

Biocides are chemicals employed to control or neutralize harmful organisms, either by direct elimination or by reducing their ability to cause damage [1]. Biocides are classified according to their intended uses and chemical characteristics, including fungicides, paraben preservatives, disinfectants, and insect repellents [2]. Currently, biocides are extensively applied in agriculture and industry, as well as cosmetics and household care products. Carbendazim (CBD), for instance, serves as a systemic fungicide with broad-spectrum efficacy, combining high potency with economic feasibility. Consequently, it is extensively applied during each harvest season in China to control fungal diseases [3]. Similarly, thiabendazole (TBD), a systemic fungicide approved for postharvest application on various fruits [4], is widely used after the fruits are harvested, before storage, transport, and sale. In personal-care products, climbazole (CLI) functions chiefly as an antifungal agent and an active constituent in dandruff-control shampoos, with a permitted level of 2.0% [5]. Similarly, other antifungal agents such as fluconazole (FCZ), clotrimazole (CTZ), and miconazole (MCZ) are primarily used to treat candidiasis via topical, oral, or intravenous administration. The widespread use of these substances leads to the release of active ingredients into the environment. For instance, approximately 80% of FCZ is excreted unchanged into the environment [6]. Topical applications can also contribute significantly to environmental loads, particularly when applied over large skin areas [7]. And these compounds were included in the European Union’s Watch List mechanism several years ago to better monitor and control their environmental presence [8]. Methylparaben (MP), due to its high stability and potent antimicrobial properties, has been widely utilized in the cosmetics industry, pharmaceuticals, and as a food preservative [9,10].

Most municipal wastewater treatment plants (WWTPs) primarily focus on the removal of inorganic nitrogen and phosphorus, but showed low removal of many biocides [11]. This deficiency may lead to the release of biocides into receiving surface water (e.g., lakes) via WWTPs effluents. Studies have demonstrated the ubiquitous occurrence of biocides in aquatic environments worldwide. Specifically, CBD has been detected in 100% of samples from the Huangpu River [12] and Yangtze River basins in China [13], surface waters in Argentina [14], and lakes/rivers in northern Vietnam [15], with maximum concentrations reaches the level of 135–369 ng/L. CLI has been detected in aquatic environments across multiple regions, including the Dongjiang River basin in China [16], the metropolitan area of Rome in Italy [17], and various rivers in Argentina [14]. MP was detected in the Yangtze River basin ranging from 18.0 to 28.8 ng/L [18], and reached 1.06 μg/L in the Xiangjiang River basin [19] and 3.62 μg/L in surface waters of Sydney, Australia [20].

Discharge of biocides into natural waters may result in adverse effects on aquatic communities and human health. For instance, CBD has been reported to exhibit embryotoxicity, reproductive toxicity, developmental toxicity, and hematological toxicity in various animal models, including humans, zebrafish (*Danio rerio*), and mice (*Mus musculus*) [21]. At 48 h post-fertilization in zebrafish, larval phenotypic abnormalities, including delayed hatching, spinal axial malformations, and pericardial edema, have been observed; moreover, embryotoxic effects have been found to vary with prolonged exposure duration [22]. Additionally, exposure to CBD has been shown to induce DNA damage in *Daphnia magna*, with a median lethal concentration (LC_50_) of only 87.6 μg/L [23]. Exposure to CLI in aquatic species can induce developmental defects, endocrine disorders, and intestinal dysbiosis; it exhibits exceptionally high toxicity to algae, with a median effective concentration (EC_50_) of 154 μg/L for *Navicula pelliculosa* [5]. High concentrations of FCZ have been found to exert cardiotoxic effects on zebrafish during cardiovascular development, with significant structural alterations of the heart and reduced heart rate [24]. Epidemiological studies suggest that MP exposure correlates with a higher incidence of gestational diabetes mellitus [25] and a shortened menstrual cycle [26]. MP has been identified as a potential developmental toxicant and metabolic disruptor in aquatic organisms, showing teratogenic effects in zebrafish larvae and alterations of lipid metabolic pathways in the blood, hepatic tissue, and intestine of adults, thereby posing ecological risks [27].

Chao Lake, located in eastern China, is a typical large shallow freshwater lake characterized by a broad surface area (464 km^2^), limited mean depth (1.64 m), and substantial water storage capacity (760 million m^3^) [28]. Studies have shown that Chao Lake is also a freshwater body under multiple pollution stresses [29,30]. Chao Lake has historically played a crucial role in social and economic functions, providing a reliable water source for over 9.6 million people. However, the deterioration of the lake ecosystem, declining water quality, contamination by organic micropollutants, and significant reductions in fish populations and other aquatic organisms have raised increasing concerns [31,32,33]. In addition to the above ecological issues, various emerging contaminants, such as perfluoroalkyl substances [28,34,35], polycyclic aromatic hydrocarbons [36], and antibiotics [37], have been detected in the water, sediments, and biota of Chao Lake, where they can migrate, accumulate, and pose potential ecological risks. Currently, research on the contamination characteristics of commonly used biocides in the aquatic environment of Chao Lake, the consequent risks to ecosystems and human health are still scarce.

To address this gap, a total of 27 sampling points were established across Chao Lake and its tributaries to conduct a comprehensive survey and assessment of biocide contamination. This approach enabled the characterization of biocide pollution profiles and the evaluation of current ecological and human health risk levels, providing a basis for recommendations to reduce biocide ecotoxicity and human health risk.

## 2. Materials and Methods

### 2.1. Materials and Equipment

In this study, seven target compounds were selected, including CBD, TBD, FCZ, CTZ, CLI, MCZ, and MP. The isotopically labeled internal standards used were thiabendazole-D_6_, fluconazole-D_4_, clotrimazole-D_5_, climbazole-D_5_, and methylparaben-D_4_. Methanol and other HPLC grade reagents were purchased from Merck and CNW Technologies. Table S1 provides detailed information on the target compounds employed in this work. In addition, Oasis HLB cartridges (Waters, Milford, MA, USA) and GF/F glass fiber filters with a 0.7 μm pore size (Whatman, Maidstone, UK) were employed in the analysis.

### 2.2. Sample Collection

**Figure 1:**
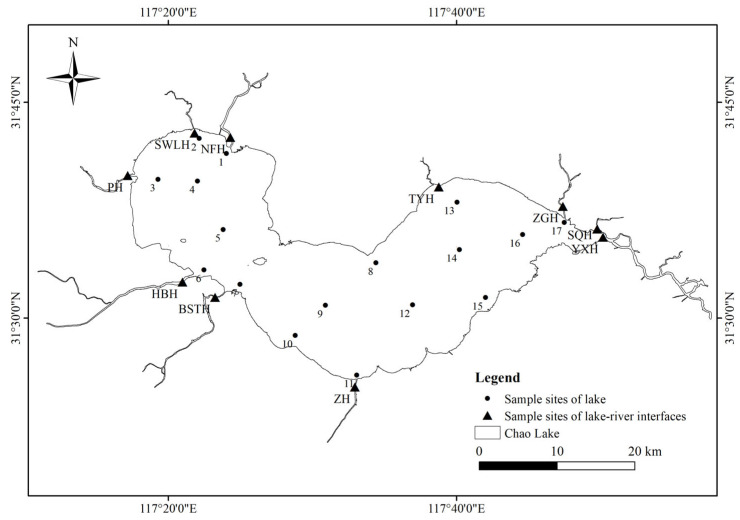
The map of sampling sites in Chao Lake and its tributaries.

The water samples at 27 sampling sites were collected from Chao Lake and its tributaries in August 2023, as illustrated in Figure 1. Based on geographical location, three regions were delineated in the lake: western (C1-C7), central (C8-C11), and eastern (C12-C17). The tributaries included Yuxi River (YXH), Shuangqiao River (SQH), Baishitian River (BSTH), Zhao River (ZH), Pai River (PH), Hangbu River (HBH), Zhegao River (ZGH), Tongyang River (TYH), Shiwuli River (SWLH), and Nanfei River (NFH). Chao Lake serves as an important freshwater resource for both Hefei and Chaohu cities. Due to the dense urban population and many factories, the inflow area situated in the western part of the lake has become a major sink for pollutants. During sampling, surface water was collected using an acrylic water sampler at a depth of 0.5 m below the water surface. The sampling sites were randomly distributed across different functional zones of the lake, covering central, nearshore, and tributaries areas, to ensure spatial representativeness. To preserve the samples, 50 mL of analytical-grade methanol and 4 M sulfuric acid were introduced onsite immediately after collection to adjust the pH to 3. The samples were stored at 4 °C and subjected to analysis within 48 h after collection. The water quality parameters of the water samples were measured following the specifications of the lake eutrophication survey [38], and details on the sampling sites, sampling dates, and physicochemical parameters are provided in Table S2.

### 2.3. Sample Extraction

Biocides in the samples were extracted using solid-phase extraction (SPE). In short, each water sample was initially filtered using a 0.7 μm GF/F membrane to eliminate suspended solids, after which 100 μL of 1 ppm internal standard mixture were added and thoroughly mixed. Before sample loading, HLB cartridges were pretreated with 10 mL methanol followed by 10 mL ultrapure water. Samples were subsequently loaded onto the SPE cartridges at a flow rate of 5–10 mL/min. Finally, the cartridge was dried under vacuum for 3 h. The target compounds were eluted into glass vials using 12 mL methanol. The eluates were dried and concentrated under a gentle nitrogen stream, then redissolved in 1 mL methanol, and finally filtered through a 0.22 μm membrane filter into a 2 mL amber glass vial. The filtered extracts were frozen at −18 °C until further instrumental analysis.

### 2.4. Instrumental Analysis and Quality Control

Analysis of the target biocides was performed using an Agilent 1200 series UPLC system combined with an Agilent 6460 triple quadrupole mass spectrometer equipped with electrospray ionization operated in both positive and negative modes (UPLC-ESI-MS/MS). To minimize contaminants introduced during experimental procedures, all glassware must be thoroughly cleaned and baked at 450 °C for 4 h in a muffle furnace. This study employed the internal standard method for the quantitative analysis of the target biocides. The method recoveries, limit of detection (LOD) and limit of quantification (LOQ) for each biocide are listed in Table S3, the recovery, LOD, and LOQ were 77.9–121%, 0.09–0.23 ng/L, and 0.30–0.76 ng/L, respectively.

### 2.5. Ecological Risk Assessment

The risk quotient (RQ) method was employed to assess the ecological risks posed by the biocides in surface water (Equation (1)) [39]. The predicted no-effect concentrations (PNEC) for the biocides are summarized in Table S4 [5,23,40,41,42,43,44,45,46,47]. The PNEC was derived based on the no-observed effect concentration value (NOEC). When NOEC values are available in the database, they are prioritized for PNEC calculation. Otherwise, the median effective concentration (EC_50_) or median lethal concentration (LC_50_) values are considered for estimation. See Equations (2) and (3) for the specific calculation methods. The risk assessment incorporated three trophic levels, including fish, aquatic invertebrates, and algae. The formula for the calculation is given below:

*RQ* = *MEC*/*PNEC*, (1)

In the equation, MEC refers to the actual concentration of a compound detected in aquatic environments, while PNEC denotes the predicted no-effect concentration. According to the magnitude of the RQ value, the ecological risk to aquatic organisms is classified as follows: RQ ≤ 0.01 indicates negligible risk; 0.01 < RQ < 0.1 corresponds to low risk; 0.1 ≤ RQ < 1 represents moderate risk; and RQ ≥ 1 denotes high risk [48].

*PNEC* = *LC*_50_ (*EC*_50_)/*AF*, (2)

*PNEC* = *NOEC*/*AF*, (3)

When at least one LC_50_ or EC_50_ value is available for any of the three trophic levels, the assessment factor (AF) is set at 1000. If NOEC values are available for one, two, or all three trophic levels, the AF is assigned as 100, 50, or 10, respectively [18].

Considering the overall hazards of the target compounds in surface waters to aquatic communities, we employed the hazard index (HI) to represent the potential cumulative ecological risks at each sampling site (Equation (4)) [49].

*HI* = ∑*RQ_i_*, (4)

### 2.6. Human Health Risk Assessment

The human health risk assessment was expressed using the risk quotient for human health (RQ_h_) (Equation (5)), calculating as the ratio of the biocide concentration in the sample (C_S_) to the age-specific drinking water equivalent level (DWEL) under a worst-case scenario approach (Equation (6)). The selection of age groups and drinking water intake was based on the Ministry of Environmental Protection of China document “Highlights of the Chinese Exposure Factors Handbook”. An RQ_h_ value of ≥1 indicates a potential health risk, an RQ_h_ value between 0.1 and 1 suggests the need for further investigation, while an 0.01 < RQ_h_ ≤ 0.1 implies no significant impact on human health, while an RQ_h_ ≤ 0.01 indicates that the risk can be considered negligible [50].

*RQ_h_* = *C_S_*/*DWEL*, (5)

*DWEL* = *ADI* × *BW* × *HQ/DWI* × *AB* × *FOE*, (6)

In the equation, ADI refers to the acceptable daily intake (μg/kg/day), which is obtained from authoritative agencies or relevant literature. BW denotes the average body weight (kg) across different age groups [51,52]. HQ represents the hazard quotient, assumed to be 1 in this study [50]. The assumption of a hazard quotient (HQ) of 1 is a standard and conservative approach in human health risk assessment to calculate the maximum allowable concentration in drinking water for public health. DWI refers to the average daily drinking water intake (L/day) [51,52]. AB refers to the absorption rate of contaminants by the exposed population, with a gastrointestinal absorption rate assumed to be 1. FOE indicates the exposure frequency, calculated as 350 days/365 days (0.96) [50]. BW and DWI values are presented in Table S5.

If no relevant ADI data is available in the literature, the lowest observed adverse effect level (LOAEL) or the no observed adverse effect level (NOAEL) is used for calculation, applying an appropriate uncertainty factor (UF) [43]. The ADI values for CBD, CLI, and MP were obtained from the literature, while the value for FCZ was calculated in this study; all values are provided in Table S6 [43,53,54,55].

*ADI* = *LOAEL*(*NOAEL*)/*UF*. (7)

## 3. Results

### 3.1. Concentrations of Biocides in Chao Lake

**Figure 2:**
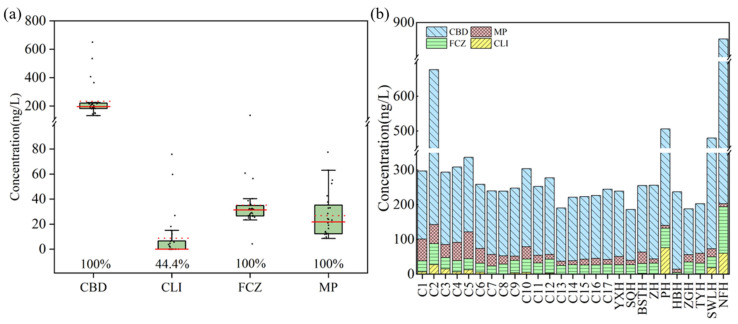
The concentration range of each biocide in surface water of Chao Lake (**a**) and the total biocide concentrations at each sampling site (**b**). The horizontal lines represent 5th, median, mean and 95th percentiles, and the boxes represent 25th and 75th percentiles. Median and mean concentrations are shown as solid and dashed horizontal lines, respectively. Biocide concentrations at each sampling site are represented by individual points. Values below the boxes are detection frequencies (%).

Four biocides including CBD, CLI, FCZ, and MP were detected, while the other four (TBD, CTZ and MCZ) were not detected in any of the sampling sites in this study (Figure 2a). The biocide levels spanned from 186 ng/L to 853 ng/L, while the corresponding mean and median were 305 and 253 ng/L, respectively. Among these, 20 sampling sites exhibited total biocide concentrations below 300 ng/L, accounting for 74.1% of the total sites. Additionally, 3 sites (11.1%) had concentrations between 300 and 500 ng/L, while 4 sites (14.8%) showed total biocide concentrations exceeding 500 ng/L.

CBD, FCZ, and MP were detected in every sampling site (100%), whereas CLI was detected at the detection frequency of 44.4%. Figure 2b and Table S7 present biocide levels in Chao Lake. The maximum level of CBD was 650 ng/L (NFH), with average and median concentration of 234 ng/L and 196 ng/L, respectively. FCZ exhibited a maximum concentration of 134 ng/L, with the mean and median values of 35.3 and 31.4 ng/L, respectively. The preservative MP showed a maximum concentration of 77.5 ng/L, with mean and median concentrations of 26.8 ng/L and 21.8 ng/L, respectively. The highest concentration of CLI was 75.8 ng/L, while its mean concentration was 8.85 ng/L.

### 3.2. Correlations Between Biocides and Environmental Variables

**Figure 3:**
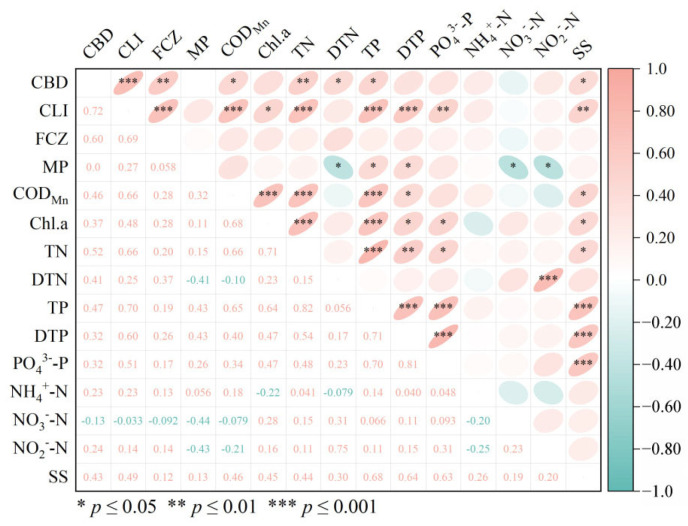
Heat maps of correlations between biocides and environmental factors. CBD: Carbendazim; CLI: Climbazole; FCZ: Fluconazole; MP: Methylparaben; COD_Mn_: permanganate index, mg/L; Chl.a: chlorophyll a, mg/L; TN: total nitrogen, mg/L; DTN: dissolved total nitrogen, mg/L; TP: total phosphorus, mg/L; DTP: dissolved total phosphorus, mg/L; PO_4_^3−^-P: orthophosphate, mg/L; NH_4_^+^-N: ammonium nitrogen, mg/L; NO_3_^−^-N: nitrate nitrogen, mg/L; NO_2_^−^-N: nitrite nitrogen, mg/L; SS: suspended solids, mg/L.

As shown in Figure 3, CBD, CLI, and FCZ displayed highly significant positive correlation (r = 0.75–0.83, *p* ≤ 0.001). However, no statistically significant relationship was found between these three biocides and MP (*p* > 0.05). The correlation analysis between the detected biocides and the water quality parameters (COD_Mn_, TN, TP, DTN, and DTP) indicated that CBD, CLI, and FCZ exhibited a highly significant correlation with DTN and NO_3_^−^-N (r = 0.67–0.83, *p* ≤ 0.001). Additionally, CBD and CLI were strongly correlated with TN (r = 0.72–0.76, *p* ≤ 0.001). Furthermore, TN exhibited a significant association with FCZ (r = 0.67, *p* ≤ 0.01).

### 3.3. Assessment of Potential Ecological Risk

**Figure 4:**
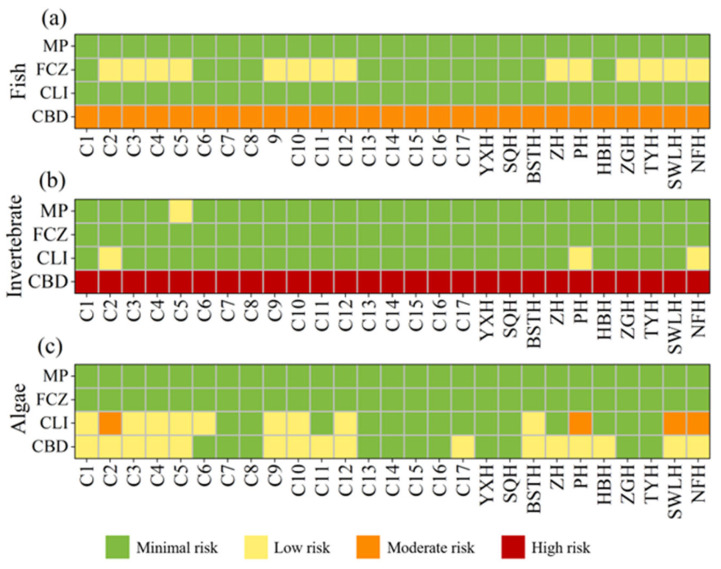
RQ values of different biocides on fish (**a**), invertebrate (**b**) and algae (**c**).

The estimated RQ values of four biocides to algae, invertebrate, and fish were summarized in Figure 4. CLI and MP exhibited RQ values far below 0.01 for fish, indicating no toxicological risk. FCZ posed a low risk to fish at 14 sampling sites, while CBD demonstrated moderate toxicity risk to fish at all sampling sites. For the invertebrate *Daphnia magna*, the RQ values of CBD ranged from 1.51 to 7.42, showing a high risk at all sampling sites. In contrast, the other three biocides posed either low or no risk to *Daphnia magna* at most sites. In the ecological risk assessment for algae, MP and FCZ posed no risk. CLI exhibited a low risk at nine sites and a moderate risk at four sites, including C2, PH, SWLH, and NFH. CBD presented a low risk to algae at 16 sampling sites.

**Figure 5:**
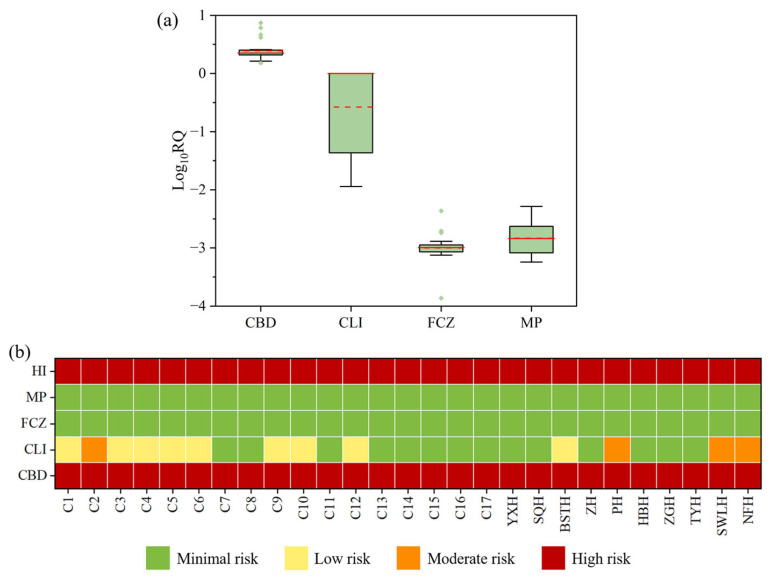
Ecological risk quotients (RQ) of the four target biocides in Chao Lake and its tributaries: (**a**) Boxplots of Log10-transformed RQs for individual biocides; (**b**) spatial distribution of RQ. The horizontal lines represent 5th, median, mean and 95th percentiles, and the boxes represent 25th and 75th percentiles. Median and mean concentrations are shown as solid and dashed horizontal lines, respectively. Outliers are displayed as individual points. Risk quotients values of different biocides. HI stands for hazard index.

The overall ecological risk assessment was conducted based on the chronic or acute toxicity data of the most sensitive species to the target compounds in surface waters (Table S4). Among the four detected biocides (CBD, CLI, FCZ, and MP), CBD exhibited an RQ value greater than 1 at all sampling sites, indicating a high ecological risk (Figure 5a,b). CLI displayed a moderate risk at four sampling sites, with RQ values between 0.1 and 1, while at other detective sites, the RQ values ranged from 0.01 to 0.1, indicating a low risk. FCZ and MP exhibited RQ values below 0.01 at all sampling sites, suggesting no significant ecological risk. The HI was employed to represent the cumulative ecological risks of the 4 biocides, with values ranging from 1.5 to 7.8, and the highest HI observed at site NFH. The results indicated that all sites in Chao Lake were at high ecological risk, with CBD identified as the major contributor.

### 3.4. Assessment of Potential Human Health Risk

**Figure 6:**
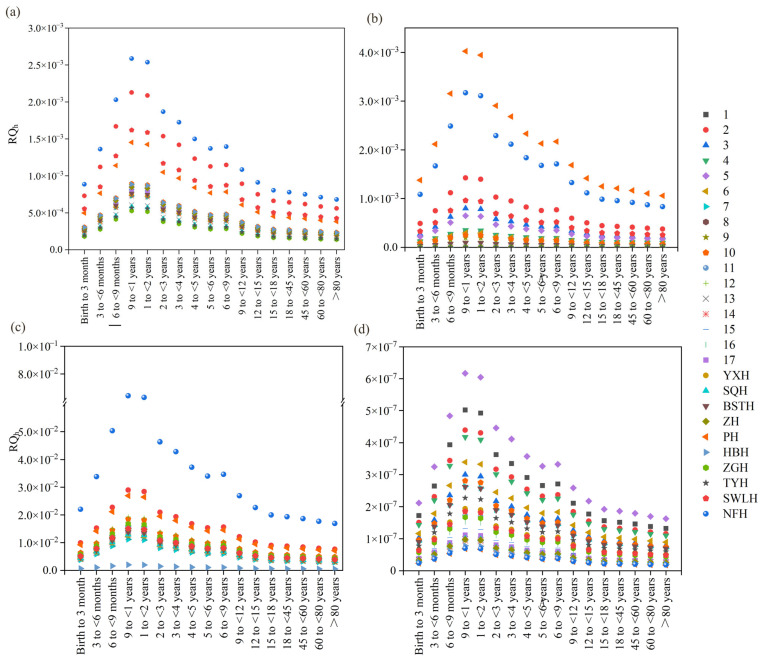
Human health risk assessment of biocides in surface water of Chao Lake: (**a**) CBD, (**b**) CLI, (**c**) FCZ, and (**d**) MP.

Human health risks of the four biocides (CBD, CLI, FCZ, and MP) were assessed using a risk evaluation model (Tables S8–S11) [50]. The results indicated that the RQ_h_ for CBD, CLI, and MP across all sampling sites and for all age groups were below 0.01, suggesting negligible human health risks (Figure 6). However, at the NFH sampling site, the RQ_h_ values of FCZ for all age groups ranged between 0.01 and 0.1, with an RQ_h_ of 0.06 observed for infants aged 9–12 months and 1–2 years (Figure 6). For infants aged 9–12 months and 1–2 years, the proportions of RQ_h_ at 0.01–0.1 for FCZ were 48.1% and 31.0%, respectively, whereas for other age groups, the proportions were below 15%. All the RO_h_ of CBD, CLL, MP and FCZ were less than 1, indicating no significant health risk.

## 4. Discussion

### 4.1. Pollution Levels of Biocides in Chao Lake

The mean concentration of CBD in the surface water of Chao Lake and its tributaries was 234 ng/L, with a detection rate of 100%. This level is comparable to the mean concentration in Tai Lake, China (223 ng/L) [56]. The median concentration (196 ng/L) was also in alignment with the median concentration reported in the Huangpu River basin during summer (200 ng/L), both exhibiting a detection frequency of 100% [12]. The concentration of CBD in Chao Lake exceeded the maximum detected concentration in the Dongjiang River basin (137 ng/L) [16] but was within the range of the maximum detected concentration in Shahe River in South China (276 ± 33.0 ng/L) [57]. Globally, CBD has been detected in surface water at varying levels. For instance, the median concentration in northern Vietnam was recorded at 86.7 ng/L [15], while in Spain’s Guadalquivir River basin, the mean concentration was reported at 159 ng/L [58]. Additionally, in Argentina’s Suquía River and Ctalamochita River, CBD was detected at multiple sites, with the highest mean concentration reaching 135 ng/L [14]. Due to its high efficacy and low application cost, CBD is one of the most widely used fungicides in China [59]. As a result, its concentration in domestic surface water systems is generally higher than those reported in international studies.

The maximum CLI concentration in Chao Lake (75.8 ng/L) was higher than that observed in the Great Lakes basin, Canada (6 ng/L) [60], but lower than the mean levels detected in the Dongjiang River basin, South China (74.8 ng/L) [16]. The mean and median concentrations of FCZ were 35.3 ng/L and 31.4 ng/L, respectively, which were consistent with the concentration range reported in the Longgang River, Shenzhen (38.1–51.6 ng/L) [61], the Dongjiang River basin, South China (45.8 ng/L) [38], and the median concentration in the Pearl River Delta (27.9 ng/L) [62]. However, these values exceeded the maximum concentration recorded in the Great Lakes basin, Canada (19 ng/L) [60] and the average concentration detected in Thailand’s coastal environment (1.52 ng/L) [63]. For MP, the mean and median concentrations were 26.8 ng/L and 21.8 ng/L, respectively, which were lower than the average concentration reported in the Dongjiang River basin, South China (46.1 ng/L) [16], the coastal environment of Thailand (59.7 ng/L) [63], the average concentration in Vietnamese lakes (51.8 ng/L) [64], and the concentration range reported in Polish rivers (36–466 ng/L) [65].

In summary, the concentration of CBD and CLI in Chao Lake are comparable to those in surface waters of other regions in China but higher than those reported in other countries. In contrast, the concentrations of other biocides, such as FCZ, and MP, are similar to or marginally lower than levels found in other domestic and international records.

### 4.2. Distribution Characteristics and Sources of Biocides

In this study, the average biocide concentrations followed the order: western region (344 ng/L) > central region (261 ng/L) > eastern region (231 ng/L). The western region exhibited a higher level of biocide contamination compared with the central and eastern regions. The upstream tributaries of the western lake basin flow through Hefei’s main urban district, Feixi County, and Feidong County, where domestic wastewater discharge via WWTPs effluents is relatively high. This could contribute to elevated emissions of azole fungicides (FCZ and CLI) and the preservative MP. Additionally, the intensified agricultural non-point source pollution in this region, combined with the passage of the NFH through an eco-agriculture tourism zone, may explain the elevated CBD in this area. The total biocide concentrations at the sites on the tributaries NFH, SWLH, and PH were 852 ng/L, 479 ng/L, and 506 ng/L, respectively. These values were higher than those observed at other river tributaries sites (186–255 ng/L), indicating that the primary sources of biocide contamination in Chao Lake are the NFH, SWLH, and PH.

Correlation analysis revealed a significant association between the concentrations of CBD, CLI, and FCZ with total nitrogen (TN) and dissolved total nitrogen (DTN) (*p* ≤ 0.01). Additionally, CBD and CLI also exhibited a correlation with total phosphorus (TP). These correlations likely indicate shared pollution sources, primarily agricultural runoff and domestic wastewater discharge, as the release of these effluents is a well-documented cause of elevated TN and TP levels in aquatic environments. Furthermore, our findings align with previous studies conducted in the Yangtze River and Dongjiang River basins [16,18], providing further evidence that the primary sources of biocide contamination in the lake are likely effluents from domestic sewage and diffuse pollution from agricultural runoff.

### 4.3. Risks and Management of Biocides

This study conducted an ecological risk assessment using RQ method. Among the detected biocides, CBD posed a moderate risk to fish, a high risk to *Daphnia magna*, and a low risk to algae. The accumulation of CBD in surface waters may exert chronic or acute toxic effects on aquatic organisms. Previous research has demonstrated that exposure to CBD can lead to developmental abnormalities in zebrafish embryos, with EC_50_ values at the mg/L level [66]. Furthermore, CBD has been demonstrated to induce multiple physiological and biochemical responses, altering the expression of numerous genes involved in apoptosis pathways, immune responses, and endocrine disruption during zebrafish embryonic development [67]. It has also been found to impact the stress response of *Daphnia magna*, causing alterations in DNA replication/repair, neurotransmission, and protein synthesis-related genes, leading to embryo toxicity, apoptosis, teratogenicity, infertility, and hematological dysfunction in other aquatic organisms [21,68,69]. For CLI, moderate to low risks to algae were observed at certain sampling sites, whereas its risks to fish and *Daphnia magna* were negligible. This is likely due to the higher toxicity of CLI to primary producers, with an EC_50_ value of 153.6 μg/L for the diatom *Navicula pelliculosa* [5].

In the human health risk assessment, the RQ_h_ values of FCZ for infants aged 9–12 months and 1–2 years exceeded 0.01 at certain sites, suggesting no significant but nonnegligible health effects. Previous studies have demonstrated that FCZ exhibits high persistence in WWTPs and is not effectively removed during conventional treatment processes [70,71,72]. Given its low removal efficiency, WWTPs should consider implementing advanced treatment technologies and optimizing existing treatment processes. Additionally, drinking water treatment plants should also prioritize enhancing the removal of FCZ to reduce its entry into the drinking water system, thereby mitigating potential health risks to infants. CBD, CLI, and MP did not exhibit potential human health risks across all age groups, which can be attributed to their relatively low toxicity to humans; instead, their adverse effects are more significant on aquatic organisms. Moreover, according to previous toxicokinetic studies, CLI does not exhibit significant genotoxic or carcinogenic potential in humans [73]. MP can be readily absorbed through the skin and gastrointestinal tract, rapidly metabolized, and subsequently excreted in the urine [74]. Importantly, no evidence of carcinogenicity, teratogenicity, or mutagenicity has been reported for MP in previous toxicological evaluations [75]. With the increasing use of biocides in China, their accumulation in the environment has become inevitable, posing potential threats to both ecological systems and human health. Therefore, appropriate measures should be implemented to minimize the input of biocides into Chao Lake as much as possible.

## 5. Conclusions

Among the 27 sampling sites in Chao Lake and its tributaries, four target biocides were detected, with the mean concentrations following the order: CBD > FCZ > MP > CLI. The highest total biocide concentration was recorded at the NFH sampling site, reaching 852.45 ng/L. In spatial distribution, western region of Chao Lake exhibited higher biocide contamination levels compared with the central and eastern regions. The concentrations of CBD, CLI, and FCZ showed highly significant positive correlations with each other, DTN and NO_3_^−^-N, while CBD and CLI were significantly associated with TN (*p* ≤ 0.001). These findings suggest that biocides CBD, CLI, and FCZ in Chao Lake may originate from domestic wastewater effluents and agricultural non-point source pollution. The RQ assessment revealed that CBD posed a high risk for invertebrates and a moderate risk for fish at all sampling sites, while CLI presented a moderate risk at certain sites. The RQ values of FCZ and MP were below 0.01 across all sampling locations, indicating negligible ecological risk. The human health risk assessment model indicated that FCZ at the NFH site posed no significant but nonnegligible impact on infants aged 9–12 months and 1–2 years, while the health risks posed by other biocides can be considered negligible. To better protect urban freshwater ecosystems, it is recommended that relevant authorities prioritize the control of CBD and CLI pollution in the Chao Lake Basin. Emphasis should be placed on strengthening the regulation of agricultural runoff and domestic sewage discharges and promoting green agricultural practices to reduce fungicide use at the source. Simultaneously, it is advised to upgrade wastewater treatment facilities in the inflow areas by implementing advanced treatment processes to effectively remove persistent biocides.

## Data Availability

The original contributions presented in this study are included in the article. Further inquiries can be directed to the corresponding authors.

## Supplementary Materials

The following supporting information can be downloaded at: https://www.mdpi.com/article/10.3390/toxics13111001/s1, Table S1: Detailed information on the target compounds; Table S2: Detailed information on the surface water in Chao Lake and its tributaries; Table S3: Recovery, detection limit and quantitation limit of target biocides in surface water; Table S4: Toxicity data, assessment factor, and predicted no-effect concentration values for the target biocides; Table S5: Average body weight and average total water intake in Chinese population groups stratified by age; Table S6: Acceptable daily intake of the detected biocides; Table S7: Concentrations of biocides in the Chao Lake and its tributaries; Table S8: RQ_h_ of the CBD in the surface water for the different age groups; Table S9: RQ_h_ of the CLI in the surface water for the different age groups; Table S10: RQ_h_ of the FCZ in the surface water for the selected age-groups; Table S11: RQ_h_ of the MP in the surface water for the different age groups.

## Abbreviations

The following abbreviations are used in this manuscript:

CBD	Carbendazim
CLI	Climbazole
MP	Methylparaben
FCZ	Fluconazole
TBD	Thiabendazole
CTZ	Clotrimazole
MCZ	Miconazole
RQ	Risk quotient
PNEC	Predicted no-effect concentrations
NOEC	No-observed effect concentration value
LC_50_	Median lethal concentration
EC_50_	Median effective concentration
AF	Assessment factor
HI	Hazard index
RQ_h_	Risk quotient for human health
WWTPs	Wastewater treatment plants
TN	Total nitrogen
DTN	Dissolved total nitrogen
TP	Total phosphorus
YXH	Yuxi River
SQH	Shuangqiao River
BSTH	Baishitian River
ZH	Zhao River
HBH	Hangbu River
ZGH	Zhegao River
TYH	Tongyang River
NFH	Nanfei River
PH	Pai River
SWLH	Shiwuli River
