# Supplementary material for: Occurrence, Distribution and Risk Assessment of Biocides in Chao Lake and Its Tributaries
Source: Toxics. 2025 Nov 20;13(11):1001. doi: 10.3390/toxics13111001 (PMC12655945; doi:10.3390/toxics13111001)
Supplement: Supplementary file 1 [file toxics-13-01001-s001.zip › toxics-3982132-supplementary.pdf]

*Supplementary Material for*

**Occurrence, Distribution and Risk Assessment of Biocides in Chao Lake and its Tributaries**

Longxiao Ji [a, b], Lei Jiang [b, e], Shengxing Wang [b, d], Xiaozhen Hu [a], Kaining Chen [b, h], Qinglong L. Wu [b, c, f, g], Li-Jun Zhou [b *]

[a] School of Environmental Science and Engineering, Nanjing University of Information Science and Technology, No.219, Ningliu Road, Nanjing 210044, China

[b] State Key Laboratory of Lake and Watershed Science for Water Security, Nanjing Institute of Geography and Limnology, Chinese Academy of Sciences, Nanjing 211135, China.

[c] Center for Evolution and Conservation Biology, Southern Marine Sciences and Engineering Guangdong Laboratory (Guangzhou), Guangzhou 511458, China

[d] School of Life Sciences, Institute of Life Science and Green Development, Hebei University, Baoding 071002, China

[e] University of Chinese Academy of Sciences, Beijing, 100049, China

[f] Sino-Danish Center for Science and Education, University of Chinese Academy of Sciences, Beijing 100039, China

[g] The Fuxianhu Station of Plateau Deep Lake Research, Chinese Academy of Sciences, Yuxi 653100, China

[h] Collaborative Innovation Center of Technology and Material of Water Treatment, Suzhou University of Science and Technology, Suzhou 215000, China

* Corresponding author：

*Email address:* ljzhou@niglas.ac.cn (Li-Jun Zhou) at State Key Laboratory of Lake and Watershed Science for Water Security, Nanjing Institute of Geography and Limnology, Chinese Academy of Sciences, Nanjing 211135, China.

**Contents:**


| **Supplementary tables** | |
|---|---|
| Table S1. Detailed information on the target compounds. | S3 |
| Table S2. Detailed information on the surface water in Chao Lake and its tributaries. | S4-S5 |
| Table S3. Recovery, detection limit and quantitation limit of target biocides in surface water. | S6 |
| Table S4. Toxicity data, assessment factor, and predicted no-effect concentration values for the target biocides. | S7 |
| Table S5. Average body weight and average total water intake in Chinese population groups stratified by age. | S8 |
| Table S6. Acceptable daily intake of the detected biocides. | S9 |
| Table S7. Concentrations of biocides in the Chao Lake and its tributaries. | S10 |
| Table S8. $RQ_h$ of the CBD in the surface water for the different age groups. | S11-S12 |
| Table S9. $RQ_h$ of the CLI in the surface water for the different age groups. | S13-S14 |
| Table S10. $RQ_h$ of the FCZ in the surface water for the selected age-groups. | S15-S16 |
| Table S11. $RQ_h$ of the MP in the surface water for the different age groups. | S17-S18 |

**Table S1.** Detailed information on the target compounds.

| Compound | Abbreviations | Supplier | M. W. [a] | CAS | Formula |
|---|---|---|---|---|---|
| Carbendazim | CBD | Dr. Ehrenstorfer | 191.19 | 10605-21-7 | $C_9H_9N_3O_2$ |
| Thiabendazole | TBD | Dr. Ehrenstorfer | 201.25 | 148-79-8 | $C_{10}H_7N_3S$ |
| Fluconazole | FCZ | Dr. Ehrenstorfer | 306.27 | 86386-73-4 | $C_{13}H_{12}F_2N_6O$ |
| Clotrimazole | CTZ | Dr. Ehrenstorfer | 344.8 | 23593-75-1 | $C_{22}H_{17}ClN_2$ |
| Climbazole | CLI | United States Pharmacopeia | 292.76 | 38083-17-9 | $C_{15}H_{17}ClN_2O_2$ |
| Miconazole | MCZ | United States Pharmacopeia | 416.1 | 22916-47-8 | $C_{18}H_{14}Cl_4N_2O$ |
| Methylparaben | MP | AccuStandard | 152.15 | 99-76-3 | $C_8H_8O_3$ |

[a] molecular weight, data from the http://www.chemspider.com/.

**Table S2.** Detailed information on the surface water in Chao Lake and its tributaries.

| Sampling sites | Sampling time | Longitude | Latitude | $COD_{Mn}$ [k] | Chl. *a* [l] | TN [m] | DTN [n] | TP [o] | DTP [p] | $PO_4^{3-}$-P [q] | $NH_4^+$-N [r] | $NO_3^-$-N [s] | $NO_2^-$-N [t] | SS [u] |
|---|---|---|---|---|---|---|---|---|---|---|---|---|---|---|
| C1 | August 2023 | 117.400353 | 31.690473 | 8.89 | 76.99 | 1.74 | 0.58 | 0.269 | 0.126 | 0.081 | 0.16 | 0.11 | 0.001 | 40.8 |
| C2 | August 2023 | 117.368928 | 31.707962 | 19.36 | 128.76 | 2.89 | 0.63 | 0.374 | 0.109 | 0.069 | 0.22 | 0.09 | 0.002 | 48.4 |
| C3 | August 2023 | 117.321200 | 31.660700 | 11.82 | 73.93 | 2.66 | 0.51 | 0.347 | 0.114 | 0.083 | 0.22 | 0.11 | 0.001 | 108.8 |
| C4 | August 2023 | 117.366904 | 31.658625 | 7.54 | 32.85 | 1.11 | 0.49 | 0.166 | 0.027 | 0.007 | 0.17 | 0.11 | 0.000 | 29.6 |
| C5 | August 2023 | 117.396610 | 31.602526 | 6.19 | 25.00 | 1.06 | 0.56 | 0.130 | 0.042 | 0.012 | 0.19 | 0.09 | 0.003 | 16.0 |
| C6 | August 2023 | 117.374449 | 31.555705 | 8.41 | 26.42 | 1.87 | 0.55 | 0.308 | 0.095 | 0.073 | 0.35 | 0.09 | 0.002 | 124.4 |
| C7 | August 2023 | 117.416240 | 31.539042 | 7.62 | 67.48 | 1.39 | 0.50 | 0.186 | 0.036 | 0.018 | 0.20 | 0.14 | 0.001 | 29.2 |
| C8 | August 2023 | 117.573319 | 31.563968 | 6.59 | 42.57 | 1.04 | 0.45 | 0.113 | 0.011 | 0.007 | 0.16 | 0.09 | 0.001 | 20.4 |
| C9 | August 2023 | 117.514929 | 31.514792 | 10.00 | 42.66 | 1.06 | 0.53 | 0.096 | 0.015 | 0.005 | 0.19 | 0.10 | 0.001 | 20.8 |
| C10 | August 2023 | 117.480000 | 31.480000 | 7.46 | 55.06 | 2.03 | 0.57 | 0.132 | 0.022 | 0.006 | 0.16 | 0.09 | 0.001 | 22.0 |
| C11 | August 2023 | 117.551158 | 31.434072 | 6.27 | 27.25 | 0.93 | 0.56 | 0.094 | 0.013 | 0.003 | 0.26 | 0.10 | 0.001 | 28.8 |
| C12 | August 2023 | 117.615816 | 31.515293 | 6.27 | 34.31 | 0.86 | 0.80 | 0.110 | 0.012 | 0.009 | 0.17 | 0.10 | 0.002 | 23.6 |
| C13 | August 2023 | 117.667400 | 31.634300 | 5.32 | 36.33 | 1.98 | 0.53 | 0.120 | 0.013 | 0.003 | 0.18 | 0.11 | 0.001 | 10.0 |
| C14 | August 2023 | 117.670146 | 31.579243 | 5.08 | 22.40 | 0.91 | 0.58 | 0.089 | 0.013 | 0.003 | 0.18 | 0.12 | 0.002 | 20.8 |
| C15 | August 2023 | 117.700170 | 31.523809 | 9.92 | 48.40 | 1.28 | 0.51 | 0.104 | 0.012 | 0.007 | 0.14 | 0.10 | 0.001 | 18.8 |
| C16 | August 2023 | 117.743243 | 31.596646 | 5.00 | 22.57 | 0.98 | 0.46 | 0.137 | 0.014 | 0.008 | 0.18 | 0.10 | 0.002 | 29.2 |
| C17 | August 2023 | 117.791380 | 31.610721 | 5.55 | 21.54 | 0.96 | 0.70 | 0.112 | 0.015 | 0.006 | 0.44 | 0.11 | 0.002 | 31.6 |
| BSTH [a] | August 2023 | 117.387389 | 31.524167 | 10.79 | 49.74 | 1.53 | 0.45 | 0.204 | 0.077 | 0.010 | 0.16 | 0.11 | 0.001 | 26.8 |
| HBH [b] | August 2023 | 117.349663 | 31.541713 | 6.35 | 52.27 | 1.97 | 1.58 | 0.132 | 0.039 | 0.029 | 0.14 | 0.59 | 0.054 | 38.8 |

| | | | | | | | | | | | | | | |
|---|---|---|---|---|---|---|---|---|---|---|---|---|---|---|
| NFH [c] | August 2023 | 117.405226 | 31.709243 | 6.90 | 59.53 | 4.48 | 2.36 | 0.209 | 0.061 | 0.015 | 0.19 | 2.09 | 0.010 | 61.2 |
| PH [d] | August 2023 | 117.286311 | 31.665063 | 16.82 | 171.69 | 4.10 | 1.59 | 0.310 | 0.031 | 0.031 | 0.37 | 0.55 | 0.061 | 44.4 |
| SWLH [e] | August 2023 | 117.363646 | 31.714368 | 54.28 | 1122.74 | 6.19 | 0.63 | 0.596 | 0.040 | 0.012 | 0.11 | 0.11 | 0.006 | 84.0 |
| SQH [f] | August 2023 | 117.829532 | 31.603186 | 5.00 | 42.72 | 0.75 | 0.60 | 0.089 | 0.025 | 0.007 | 0.11 | 0.14 | 0.004 | 31.6 |
| TYH [g] | August 2023 | 117.646286 | 31.651905 | 5.87 | 55.86 | 1.27 | 0.67 | 0.148 | 0.021 | 0.007 | 0.13 | 0.14 | 0.009 | 24.0 |
| YXH [h] | August 2023 | 117.836193 | 31.593763 | 8.01 | 43.30 | 0.92 | 0.52 | 0.128 | 0.020 | 0.006 | 0.26 | 0.09 | 0.001 | 37.2 |
| ZH [i] | August 2023 | 117.549221 | 31.420139 | 2.14 | 34.77 | 1.02 | 0.56 | 0.119 | 0.030 | 0.018 | 0.17 | 0.05 | 0.003 | 25.2 |
| ZGH [j] | August 2023 | 117.789950 | 31.629438 | 4.68 | 32.65 | 0.88 | 0.59 | 0.099 | 0.049 | 0.013 | 0.15 | 0.11 | 0.002 | 22.0 |

[a] BSTH, Baishitian River; [b] HBH, Hangbu River; [c] NFH, Nanfei River, [d] PH, Pai River; [e] SWLH, Shiwuli River; [f] SQH, Shuangqiao River; [g] TYH, Tongyang River; [h] YXH, Yuxi River; [i] ZH, Zhao River; [j] ZGH, Zhegao River; [k] $COD_{Mn}$, permanganate index; [l] Chl. *a*, chlorophyll *a*; [m] TN, total nitrogen; [n] DTN, dissolved total nitrogen; [o] TP, total phosphorus; [p] DTP, dissolved total phosphorus; [q] $PO_4^{3-}$—P, orthophosphate; [r] $NH_4^+$-N, ammonium nitrogen; [s] $NO_3^-$—N, nitrate nitrogen; [t] $NO_2^-$—N, nitrite nitrogen; [u] SS, suspended solids.

**Table S3.** Recovery, detection limit and quantitation limit of target biocides in surface water

| Biocide | Recoveries (100 ng/L, n=3) | LOD [a] (ng/L) | LOQ [b] (ng/L) |
|---|---|---|---|
| CBD | 95.3 ± 14.0 | 0.18 | 0.60 |
| TBD | 79.6± 10.5 | 0.12 | 0.41 |
| CLI | 110 ± 20.5 | 0.13 | 0.43 |
| FCZ | 77.9 ± 9.6 | 0.13 | 0.44 |
| CTZ | 121 ± 16.1 | 0.23 | 0.76 |
| MCZ | 91.8 ± 7.0 | 0.15 | 0.49 |
| MP | 102 ± 0.7 | 0.09 | 0.30 |

[a] Limit of detection (LOD); [b] Limit of quantification (LOQ).

**Table S4.** Toxicity data, assessment factor, and predicted no-effect concentration values for the target biocides

| Biocides | Trophic level | Species | Exposure duration /day | Types of toxicity data | Concentrations /(μg/L) | AF [a] | PNEC/(ng/L) [b] | References |
|---|---|---|---|---|---|---|---|---|
| CBD | Fish | *Danio rerio* | 4 | $LC_{50}$ [c] | 890 | 1000 | 87.6 | [1] |
| | Invertebrate | *Daphnia magna* | 2 | $LC_{50}$ | 87.6 | | | [2] |
| | Algae | *Scenedesmus obliqnus* | 4 | $EC_{50}$ [d] | $1.9\times10^4$ | | | [3] |
| CLI | Fish | *Danio rerio* | 2 | $EC_{50}$ | $8.2\times10^3$ | 1000 | 153.6 | [4] |
| | Invertebrate | *Daphnia similis* | 2 | $LC_{50}$ | $2.5\times10^3$ | | | [5] |
| | Algae | *Navicula pelliculosa* | 3 | $EC_{50}$ | 153.6 | | | [4] |
| FCZ | Fish | *Danio rerio* | 5 | NOEC [e] | 306 | 10 | $3.1\times10^4$ | [6] |
| | Invertebrate | *Daphnia magna* | 21 | NOEC | 3060 | | | [7] |
| | Algae | *Pseudokirchneriella subcapitata* | 3 | NOEC | $1.6\times10^4$ | | | [8] |
| MP | Fish | *Pimephales promelas* | 7 | NOEC | $1.3\times10^4$ | 10 | $7.5\times10^4$ | [9] |
| | Invertebrate | *Daphnia magna* | 10 | NOEC | 750 | | | [9] |
| | Algae | *Psuedokirchneriella subcapitata* | 3 | NOEC | $2.1\times10^4$ | | | [10] |

[a] AF, assessment factor; [b] PNEC, predicted no-effect concentration; [c] $LC_{50}$, lethal concentration 50%; [d] $EC_{50}$, effective concentration 50%; [e] NOEC, no-observed-effect concentration.

**Table S5.** Average body weight and average total water intake in Chinese population groups stratified by age.

| Age groups | Body weight (kg) | DWI (L/day) [a] |
|---|---|---|
| 0-3 months | 6.4 | 0.182 |
| 3-6 months | 7.9 | 0.345 |
| 6-9 months | 9.1 | 0.592 |
| 9-12 months | 9.8 | 0.813 |
| 1-2 years | 11.2 | 0.911 |
| 2-3 years | 13.5 | 0.809 |
| 3-4 years | 15.6 | 0.863 |
| 4-5 years | 17.7 | 0.851 |
| 5-6 years | 19.6 | 0.861 |
| 6-9 years | 26.5 | 1.186 |
| 9-12 years | 36.8 | 1.28 |
| 12-15 years | 47.3 | 1.383 |
| 15-18 years | 54.8 | 1.414 |
| 18-45 years | 61.9 | 1.547 |
| 45-60 years | 63.5 | 1.529 |
| 60-80 years | 60.3 | 1.375 |
| ⩾80 years | 55.5 | 1.212 |

[a] DWI, drinking water intakes

**Table S6.** Acceptable daily intake (ADI) of the detected biocides

| Biocides | UF [a] | LOAEL [b] | ADI (mg/kg/day) | References |
|---|---|---|---|---|
| CBD | \ [c] | \ | 0.02 | [11] |
| CLI | 30000 | 5mg/kg/day | $1.5\times10^{-3}$ | [7,12] |
| FCZ | \ | \ | $1.7\times10^{-4}$ | [7] |
| MP | \ | \ | 10 | [13] |

[a] UF, uncertainty factor; [b] LOAEL, lowest observed adverse effect level; [c] \, the ADI value was directly cited without calculation.

**Table S7.** Concentrations (ng/L) of biocides in Chao Lake.

| Sampling sites | CBD | TBD | CLI | FCZ | CTZ | MCZ | MP |
|---|---|---|---|---|---|---|---|
| C1 | 196.2 | ND [a] | 6.4 | 31.5 | ND | ND | 63.1 |
| C2 | 534.3 | ND | 26.9 | 60.7 | ND | ND | 55.1 |
| C3 | 208.3 | ND | 15.1 | 32.5 | ND | ND | 37.7 |
| C4 | 217.2 | ND | 6.7 | 32.3 | ND | ND | 52.4 |
| C5 | 214.6 | ND | 12.2 | 32.1 | ND | ND | 77.5 |
| C6 | 185.3 | ND | 5.6 | 25.5 | ND | ND | 42.6 |
| C7 | 183.3 | ND | ND | 23.4 | ND | ND | 33.1 |
| C8 | 186.5 | ND | ND | 28.8 | ND | ND | 23.8 |
| C9 | 196.4 | ND | 3.7 | 35.8 | ND | ND | 12.0 |
| C10 | 225.0 | ND | 4.7 | 38.8 | ND | ND | 35.3 |
| C11 | 198.9 | ND | ND | 32.1 | ND | ND | 21.8 |
| C12 | 221.4 | ND | 2.5 | 40.4 | ND | ND | 13.5 |
| C13 | 152.8 | ND | ND | 25.0 | ND | ND | 12.4 |
| C14 | 183.0 | ND | ND | 26.9 | ND | ND | 11.8 |
| C15 | 180.5 | ND | ND | 26.3 | ND | ND | 16.6 |
| C16 | 181.3 | ND | ND | 26.2 | ND | ND | 19.1 |
| C17 | 202.8 | ND | ND | 28.1 | ND | ND | 14.1 |
| BSTH | 188.0 | ND | ND | 26.6 | ND | ND | 24.3 |
| HBH | 146.6 | ND | ND | 27.1 | ND | ND | 12.7 |
| NFH | 191.4 | ND | 1.8 | 29.0 | ND | ND | 32.9 |
| PH | 212.6 | ND | ND | 31.4 | ND | ND | 12.3 |
| SWLH | 364.9 | ND | 75.8 | 56.4 | ND | ND | 8.6 |
| SQH | 222.9 | ND | ND | 4.3 | ND | ND | 9.8 |
| TYH | 132.5 | ND | ND | 35.0 | ND | ND | 21.0 |
| YXH | 142.9 | ND | ND | 31.4 | ND | ND | 28.5 |
| ZH | 406.5 | ND | 18.1 | 31.5 | ND | ND | 23.2 |
| ZGH | 649.6 | ND | 59.7 | 134.4 | ND | ND | 8.8 |

[a] ND: not detected.

**Table S8.** $RQ_h$ of the CBD in the surface water for the different age groups.

| Sampling sites | 0-3 months | 3-6 months | 6-9 months | 9-12 months | 1-2 years | 2-3 years | 3-4 years | 4-5 years | 5-6 years | 6-9 years | 9-12 years | 12-15 years | 15-18 years | 18-45 years | 45-60 years | 60-80 years | ≥80 years |
|---|---|---|---|---|---|---|---|---|---|---|---|---|---|---|---|---|---|
| C1 | 0.00 | 0.00 | 0.00 | 0.00 | 0.00 | 0.00 | 0.00 | 0.00 | 0.00 | 0.00 | 0.00 | 0.00 | 0.00 | 0.00 | 0.00 | 0.00 | 0.00 |
| C2 | 0.00 | 0.00 | 0.00 | 0.00 | 0.00 | 0.00 | 0.00 | 0.00 | 0.00 | 0.00 | 0.00 | 0.00 | 0.00 | 0.00 | 0.00 | 0.00 | 0.00 |
| C3 | 0.00 | 0.00 | 0.00 | 0.00 | 0.00 | 0.00 | 0.00 | 0.00 | 0.00 | 0.00 | 0.00 | 0.00 | 0.00 | 0.00 | 0.00 | 0.00 | 0.00 |
| C4 | 0.00 | 0.00 | 0.00 | 0.00 | 0.00 | 0.00 | 0.00 | 0.00 | 0.00 | 0.00 | 0.00 | 0.00 | 0.00 | 0.00 | 0.00 | 0.00 | 0.00 |
| C5 | 0.00 | 0.00 | 0.00 | 0.00 | 0.00 | 0.00 | 0.00 | 0.00 | 0.00 | 0.00 | 0.00 | 0.00 | 0.00 | 0.00 | 0.00 | 0.00 | 0.00 |
| C6 | 0.00 | 0.00 | 0.00 | 0.00 | 0.00 | 0.00 | 0.00 | 0.00 | 0.00 | 0.00 | 0.00 | 0.00 | 0.00 | 0.00 | 0.00 | 0.00 | 0.00 |
| C7 | 0.00 | 0.00 | 0.00 | 0.00 | 0.00 | 0.00 | 0.00 | 0.00 | 0.00 | 0.00 | 0.00 | 0.00 | 0.00 | 0.00 | 0.00 | 0.00 | 0.00 |
| C8 | 0.00 | 0.00 | 0.00 | 0.00 | 0.00 | 0.00 | 0.00 | 0.00 | 0.00 | 0.00 | 0.00 | 0.00 | 0.00 | 0.00 | 0.00 | 0.00 | 0.00 |
| C9 | 0.00 | 0.00 | 0.00 | 0.00 | 0.00 | 0.00 | 0.00 | 0.00 | 0.00 | 0.00 | 0.00 | 0.00 | 0.00 | 0.00 | 0.00 | 0.00 | 0.00 |
| C10 | 0.00 | 0.00 | 0.00 | 0.00 | 0.00 | 0.00 | 0.00 | 0.00 | 0.00 | 0.00 | 0.00 | 0.00 | 0.00 | 0.00 | 0.00 | 0.00 | 0.00 |
| C11 | 0.00 | 0.00 | 0.00 | 0.00 | 0.00 | 0.00 | 0.00 | 0.00 | 0.00 | 0.00 | 0.00 | 0.00 | 0.00 | 0.00 | 0.00 | 0.00 | 0.00 |
| C12 | 0.00 | 0.00 | 0.00 | 0.00 | 0.00 | 0.00 | 0.00 | 0.00 | 0.00 | 0.00 | 0.00 | 0.00 | 0.00 | 0.00 | 0.00 | 0.00 | 0.00 |
| C13 | 0.00 | 0.00 | 0.00 | 0.00 | 0.00 | 0.00 | 0.00 | 0.00 | 0.00 | 0.00 | 0.00 | 0.00 | 0.00 | 0.00 | 0.00 | 0.00 | 0.00 |

| | | | | | | | | | | | | | | | | | |
|---|---|---|---|---|---|---|---|---|---|---|---|---|---|---|---|---|---|
| C14 | 0.00 | 0.00 | 0.00 | 0.00 | 0.00 | 0.00 | 0.00 | 0.00 | 0.00 | 0.00 | 0.00 | 0.00 | 0.00 | 0.00 | 0.00 | 0.00 | 0.00 |
| C15 | 0.00 | 0.00 | 0.00 | 0.00 | 0.00 | 0.00 | 0.00 | 0.00 | 0.00 | 0.00 | 0.00 | 0.00 | 0.00 | 0.00 | 0.00 | 0.00 | 0.00 |
| C16 | 0.00 | 0.00 | 0.00 | 0.00 | 0.00 | 0.00 | 0.00 | 0.00 | 0.00 | 0.00 | 0.00 | 0.00 | 0.00 | 0.00 | 0.00 | 0.00 | 0.00 |
| C17 | 0.00 | 0.00 | 0.00 | 0.00 | 0.00 | 0.00 | 0.00 | 0.00 | 0.00 | 0.00 | 0.00 | 0.00 | 0.00 | 0.00 | 0.00 | 0.00 | 0.00 |
| YXH | 0.00 | 0.00 | 0.00 | 0.00 | 0.00 | 0.00 | 0.00 | 0.00 | 0.00 | 0.00 | 0.00 | 0.00 | 0.00 | 0.00 | 0.00 | 0.00 | 0.00 |
| SQH | 0.00 | 0.00 | 0.00 | 0.00 | 0.00 | 0.00 | 0.00 | 0.00 | 0.00 | 0.00 | 0.00 | 0.00 | 0.00 | 0.00 | 0.00 | 0.00 | 0.00 |
| BSTH | 0.00 | 0.00 | 0.00 | 0.00 | 0.00 | 0.00 | 0.00 | 0.00 | 0.00 | 0.00 | 0.00 | 0.00 | 0.00 | 0.00 | 0.00 | 0.00 | 0.00 |
| ZH | 0.00 | 0.00 | 0.00 | 0.00 | 0.00 | 0.00 | 0.00 | 0.00 | 0.00 | 0.00 | 0.00 | 0.00 | 0.00 | 0.00 | 0.00 | 0.00 | 0.00 |
| PH | 0.00 | 0.00 | 0.00 | 0.00 | 0.00 | 0.00 | 0.00 | 0.00 | 0.00 | 0.00 | 0.00 | 0.00 | 0.00 | 0.00 | 0.00 | 0.00 | 0.00 |
| HBH | 0.00 | 0.00 | 0.00 | 0.00 | 0.00 | 0.00 | 0.00 | 0.00 | 0.00 | 0.00 | 0.00 | 0.00 | 0.00 | 0.00 | 0.00 | 0.00 | 0.00 |
| ZGH | 0.00 | 0.00 | 0.00 | 0.00 | 0.00 | 0.00 | 0.00 | 0.00 | 0.00 | 0.00 | 0.00 | 0.00 | 0.00 | 0.00 | 0.00 | 0.00 | 0.00 |
| TYH | 0.00 | 0.00 | 0.00 | 0.00 | 0.00 | 0.00 | 0.00 | 0.00 | 0.00 | 0.00 | 0.00 | 0.00 | 0.00 | 0.00 | 0.00 | 0.00 | 0.00 |
| SWLH | 0.00 | 0.00 | 0.00 | 0.00 | 0.00 | 0.00 | 0.00 | 0.00 | 0.00 | 0.00 | 0.00 | 0.00 | 0.00 | 0.00 | 0.00 | 0.00 | 0.00 |
| NFH | 0.00 | 0.00 | 0.00 | 0.00 | 0.00 | 0.00 | 0.00 | 0.00 | 0.00 | 0.00 | 0.00 | 0.00 | 0.00 | 0.00 | 0.00 | 0.00 | 0.00 |

**Table S9.** $RQ_h$ of the CLI in the surface water for the different age groups.

| Sampling sites | 0-3 months | 3-6 months | 6-9 months | 9-12 months | 1-2 years | 2-3 years | 3-4 years | 4-5 years | 5-6 years | 6-9 years | 9-12 years | 12-15 years | 15-18 years | 18-45 years | 45-60 years | 60-80 years | ≥80 years |
|---|---|---|---|---|---|---|---|---|---|---|---|---|---|---|---|---|---|
| C1 | 0.00 | 0.00 | 0.00 | 0.00 | 0.00 | 0.00 | 0.00 | 0.00 | 0.00 | 0.00 | 0.00 | 0.00 | 0.00 | 0.00 | 0.00 | 0.00 | 0.00 |
| C2 | 0.00 | 0.00 | 0.00 | 0.00 | 0.00 | 0.00 | 0.00 | 0.00 | 0.00 | 0.00 | 0.00 | 0.00 | 0.00 | 0.00 | 0.00 | 0.00 | 0.00 |
| C3 | 0.00 | 0.00 | 0.00 | 0.00 | 0.00 | 0.00 | 0.00 | 0.00 | 0.00 | 0.00 | 0.00 | 0.00 | 0.00 | 0.00 | 0.00 | 0.00 | 0.00 |
| C4 | 0.00 | 0.00 | 0.00 | 0.00 | 0.00 | 0.00 | 0.00 | 0.00 | 0.00 | 0.00 | 0.00 | 0.00 | 0.00 | 0.00 | 0.00 | 0.00 | 0.00 |
| C5 | 0.00 | 0.00 | 0.00 | 0.00 | 0.00 | 0.00 | 0.00 | 0.00 | 0.00 | 0.00 | 0.00 | 0.00 | 0.00 | 0.00 | 0.00 | 0.00 | 0.00 |
| C6 | 0.00 | 0.00 | 0.00 | 0.00 | 0.00 | 0.00 | 0.00 | 0.00 | 0.00 | 0.00 | 0.00 | 0.00 | 0.00 | 0.00 | 0.00 | 0.00 | 0.00 |
| C7 | 0.00 | 0.00 | 0.00 | 0.00 | 0.00 | 0.00 | 0.00 | 0.00 | 0.00 | 0.00 | 0.00 | 0.00 | 0.00 | 0.00 | 0.00 | 0.00 | 0.00 |
| C8 | 0.00 | 0.00 | 0.00 | 0.00 | 0.00 | 0.00 | 0.00 | 0.00 | 0.00 | 0.00 | 0.00 | 0.00 | 0.00 | 0.00 | 0.00 | 0.00 | 0.00 |
| C9 | 0.00 | 0.00 | 0.00 | 0.00 | 0.00 | 0.00 | 0.00 | 0.00 | 0.00 | 0.00 | 0.00 | 0.00 | 0.00 | 0.00 | 0.00 | 0.00 | 0.00 |
| C10 | 0.00 | 0.00 | 0.00 | 0.00 | 0.00 | 0.00 | 0.00 | 0.00 | 0.00 | 0.00 | 0.00 | 0.00 | 0.00 | 0.00 | 0.00 | 0.00 | 0.00 |
| C11 | 0.00 | 0.00 | 0.00 | 0.00 | 0.00 | 0.00 | 0.00 | 0.00 | 0.00 | 0.00 | 0.00 | 0.00 | 0.00 | 0.00 | 0.00 | 0.00 | 0.00 |
| C12 | 0.00 | 0.00 | 0.00 | 0.00 | 0.00 | 0.00 | 0.00 | 0.00 | 0.00 | 0.00 | 0.00 | 0.00 | 0.00 | 0.00 | 0.00 | 0.00 | 0.00 |
| C13 | 0.00 | 0.00 | 0.00 | 0.00 | 0.00 | 0.00 | 0.00 | 0.00 | 0.00 | 0.00 | 0.00 | 0.00 | 0.00 | 0.00 | 0.00 | 0.00 | 0.00 |
| C14 | 0.00 | 0.00 | 0.00 | 0.00 | 0.00 | 0.00 | 0.00 | 0.00 | 0.00 | 0.00 | 0.00 | 0.00 | 0.00 | 0.00 | 0.00 | 0.00 | 0.00 |

| | | | | | | | | | | | | | | | | | |
|---|---|---|---|---|---|---|---|---|---|---|---|---|---|---|---|---|---|
| C15 | 0.00 | 0.00 | 0.00 | 0.00 | 0.00 | 0.00 | 0.00 | 0.00 | 0.00 | 0.00 | 0.00 | 0.00 | 0.00 | 0.00 | 0.00 | 0.00 | 0.00 |
| C16 | 0.00 | 0.00 | 0.00 | 0.00 | 0.00 | 0.00 | 0.00 | 0.00 | 0.00 | 0.00 | 0.00 | 0.00 | 0.00 | 0.00 | 0.00 | 0.00 | 0.00 |
| C17 | 0.00 | 0.00 | 0.00 | 0.00 | 0.00 | 0.00 | 0.00 | 0.00 | 0.00 | 0.00 | 0.00 | 0.00 | 0.00 | 0.00 | 0.00 | 0.00 | 0.00 |
| YXH | 0.00 | 0.00 | 0.00 | 0.00 | 0.00 | 0.00 | 0.00 | 0.00 | 0.00 | 0.00 | 0.00 | 0.00 | 0.00 | 0.00 | 0.00 | 0.00 | 0.00 |
| SQH | 0.00 | 0.00 | 0.00 | 0.00 | 0.00 | 0.00 | 0.00 | 0.00 | 0.00 | 0.00 | 0.00 | 0.00 | 0.00 | 0.00 | 0.00 | 0.00 | 0.00 |
| BSTH | 0.00 | 0.00 | 0.00 | 0.00 | 0.00 | 0.00 | 0.00 | 0.00 | 0.00 | 0.00 | 0.00 | 0.00 | 0.00 | 0.00 | 0.00 | 0.00 | 0.00 |
| ZH | 0.00 | 0.00 | 0.00 | 0.00 | 0.00 | 0.00 | 0.00 | 0.00 | 0.00 | 0.00 | 0.00 | 0.00 | 0.00 | 0.00 | 0.00 | 0.00 | 0.00 |
| PH | 0.00 | 0.00 | 0.00 | 0.00 | 0.00 | 0.00 | 0.00 | 0.00 | 0.00 | 0.00 | 0.00 | 0.00 | 0.00 | 0.00 | 0.00 | 0.00 | 0.00 |
| HBH | 0.00 | 0.00 | 0.00 | 0.00 | 0.00 | 0.00 | 0.00 | 0.00 | 0.00 | 0.00 | 0.00 | 0.00 | 0.00 | 0.00 | 0.00 | 0.00 | 0.00 |
| ZGH | 0.00 | 0.00 | 0.00 | 0.00 | 0.00 | 0.00 | 0.00 | 0.00 | 0.00 | 0.00 | 0.00 | 0.00 | 0.00 | 0.00 | 0.00 | 0.00 | 0.00 |
| TYH | 0.00 | 0.00 | 0.00 | 0.00 | 0.00 | 0.00 | 0.00 | 0.00 | 0.00 | 0.00 | 0.00 | 0.00 | 0.00 | 0.00 | 0.00 | 0.00 | 0.00 |
| SWLH | 0.00 | 0.00 | 0.00 | 0.00 | 0.00 | 0.00 | 0.00 | 0.00 | 0.00 | 0.00 | 0.00 | 0.00 | 0.00 | 0.00 | 0.00 | 0.00 | 0.00 |
| NFH | 0.00 | 0.00 | 0.00 | 0.00 | 0.00 | 0.00 | 0.00 | 0.00 | 0.00 | 0.00 | 0.00 | 0.00 | 0.00 | 0.00 | 0.00 | 0.00 | 0.00 |

**Table S10.** $RQ_h$ of FCZ in the surface water for the different age groups**.**

| Sampling sites | 0-3 months | 3-6 months | 6-9 months | 9-12 months | 1-2 years | 2-3 years | 3-4 years | 4-5 years | 5-6 years | 6-9 years | 9-12 years | 12-15 years | 15-18 years | 18-45 years | 45-60 years | 60-80 years | ≥80 years |
|---|---|---|---|---|---|---|---|---|---|---|---|---|---|---|---|---|---|
| C1 | 0.01 | 0.01 | 0.01 | 0.02 | 0.01 | 0.01 | 0.01 | 0.01 | 0.01 | 0.01 | 0.01 | 0.01 | 0.00 | 0.00 | 0.00 | 0.00 | 0.00 |
| C2 | 0.01 | 0.02 | 0.02 | 0.03 | 0.03 | 0.02 | 0.02 | 0.02 | 0.02 | 0.02 | 0.01 | 0.01 | 0.01 | 0.01 | 0.01 | 0.01 | 0.01 |
| C3 | 0.01 | 0.01 | 0.01 | 0.02 | 0.02 | 0.01 | 0.01 | 0.01 | 0.01 | 0.01 | 0.01 | 0.01 | 0.00 | 0.00 | 0.00 | 0.00 | 0.00 |
| C4 | 0.01 | 0.01 | 0.01 | 0.02 | 0.02 | 0.01 | 0.01 | 0.01 | 0.01 | 0.01 | 0.01 | 0.01 | 0.00 | 0.00 | 0.00 | 0.00 | 0.00 |
| C5 | 0.01 | 0.01 | 0.01 | 0.02 | 0.02 | 0.01 | 0.01 | 0.01 | 0.01 | 0.01 | 0.01 | 0.01 | 0.00 | 0.00 | 0.00 | 0.00 | 0.00 |
| C6 | 0.00 | 0.01 | 0.01 | 0.01 | 0.01 | 0.01 | 0.01 | 0.01 | 0.01 | 0.01 | 0.01 | 0.00 | 0.00 | 0.00 | 0.00 | 0.00 | 0.00 |
| C7 | 0.00 | 0.01 | 0.01 | 0.01 | 0.01 | 0.01 | 0.01 | 0.01 | 0.01 | 0.01 | 0.00 | 0.00 | 0.00 | 0.00 | 0.00 | 0.00 | 0.00 |
| C8 | 0.00 | 0.01 | 0.01 | 0.01 | 0.01 | 0.01 | 0.01 | 0.01 | 0.01 | 0.01 | 0.01 | 0.00 | 0.00 | 0.00 | 0.00 | 0.00 | 0.00 |
| C9 | 0.01 | 0.01 | 0.01 | 0.02 | 0.02 | 0.01 | 0.01 | 0.01 | 0.01 | 0.01 | 0.01 | 0.01 | 0.01 | 0.01 | 0.00 | 0.00 | 0.00 |
| C10 | 0.01 | 0.01 | 0.01 | 0.02 | 0.02 | 0.01 | 0.01 | 0.01 | 0.01 | 0.01 | 0.01 | 0.01 | 0.01 | 0.01 | 0.01 | 0.01 | 0.00 |
| C11 | 0.01 | 0.01 | 0.01 | 0.02 | 0.02 | 0.01 | 0.01 | 0.01 | 0.01 | 0.01 | 0.01 | 0.01 | 0.00 | 0.00 | 0.00 | 0.00 | 0.00 |
| C12 | 0.01 | 0.01 | 0.02 | 0.02 | 0.02 | 0.01 | 0.01 | 0.01 | 0.01 | 0.01 | 0.01 | 0.01 | 0.01 | 0.01 | 0.01 | 0.01 | 0.01 |
| C13 | 0.00 | 0.01 | 0.01 | 0.01 | 0.01 | 0.01 | 0.01 | 0.01 | 0.01 | 0.01 | 0.00 | 0.00 | 0.00 | 0.00 | 0.00 | 0.00 | 0.00 |
| C14 | 0.00 | 0.01 | 0.01 | 0.01 | 0.01 | 0.01 | 0.01 | 0.01 | 0.01 | 0.01 | 0.01 | 0.00 | 0.00 | 0.00 | 0.00 | 0.00 | 0.00 |

| | | | | | | | | | | | | | | | | | |
|---|---|---|---|---|---|---|---|---|---|---|---|---|---|---|---|---|---|
| C15 | 0.00 | 0.01 | 0.01 | 0.01 | 0.01 | 0.01 | 0.01 | 0.01 | 0.01 | 0.01 | 0.01 | 0.00 | 0.00 | 0.00 | 0.00 | 0.00 | 0.00 |
| C16 | 0.00 | 0.01 | 0.01 | 0.01 | 0.01 | 0.01 | 0.01 | 0.01 | 0.01 | 0.01 | 0.01 | 0.00 | 0.00 | 0.00 | 0.00 | 0.00 | 0.00 |
| C17 | 0.00 | 0.01 | 0.01 | 0.01 | 0.01 | 0.01 | 0.01 | 0.01 | 0.01 | 0.01 | 0.01 | 0.00 | 0.00 | 0.00 | 0.00 | 0.00 | 0.00 |
| YXH | 0.00 | 0.01 | 0.01 | 0.01 | 0.01 | 0.01 | 0.01 | 0.01 | 0.01 | 0.01 | 0.01 | 0.00 | 0.00 | 0.00 | 0.00 | 0.00 | 0.00 |
| SQH | 0.00 | 0.01 | 0.01 | 0.01 | 0.01 | 0.01 | 0.01 | 0.01 | 0.01 | 0.01 | 0.01 | 0.00 | 0.00 | 0.00 | 0.00 | 0.00 | 0.00 |
| BSTH | 0.00 | 0.01 | 0.01 | 0.01 | 0.01 | 0.01 | 0.01 | 0.01 | 0.01 | 0.01 | 0.01 | 0.00 | 0.00 | 0.00 | 0.00 | 0.00 | 0.00 |
| ZH | 0.01 | 0.01 | 0.01 | 0.02 | 0.01 | 0.01 | 0.01 | 0.01 | 0.01 | 0.01 | 0.01 | 0.01 | 0.00 | 0.00 | 0.00 | 0.00 | 0.00 |
| PH | 0.01 | 0.01 | 0.02 | 0.03 | 0.03 | 0.02 | 0.02 | 0.02 | 0.01 | 0.01 | 0.01 | 0.01 | 0.01 | 0.01 | 0.01 | 0.01 | 0.01 |
| HBH | 0.00 | 0.00 | 0.00 | 0.00 | 0.00 | 0.00 | 0.00 | 0.00 | 0.00 | 0.00 | 0.00 | 0.00 | 0.00 | 0.00 | 0.00 | 0.00 | 0.00 |
| ZGH | 0.01 | 0.01 | 0.01 | 0.02 | 0.02 | 0.01 | 0.01 | 0.01 | 0.01 | 0.01 | 0.01 | 0.01 | 0.01 | 0.01 | 0.00 | 0.00 | 0.00 |
| TYH | 0.01 | 0.01 | 0.01 | 0.01 | 0.01 | 0.01 | 0.01 | 0.01 | 0.01 | 0.01 | 0.01 | 0.01 | 0.00 | 0.00 | 0.00 | 0.00 | 0.00 |
| SWLH | 0.01 | 0.01 | 0.01 | 0.02 | 0.01 | 0.01 | 0.01 | 0.01 | 0.01 | 0.01 | 0.01 | 0.01 | 0.00 | 0.00 | 0.00 | 0.00 | 0.00 |
| NFH | 0.02 | 0.03 | 0.05 | 0.06 | 0.06 | 0.05 | 0.04 | 0.04 | 0.03 | 0.03 | 0.03 | 0.02 | 0.02 | 0.02 | 0.02 | 0.02 | 0.02 |

**Table S11.** $RQ_h$ of MP in the surface water for the different age groups.

| Sampling sites | 0-3 months | 3-6 months | 6-9 months | 9-12 months | 1-2 years | 2-3 years | 3-4 years | 4-5 years | 5-6 years | 6-9 years | 9-12 years | 12-15 years | 15-18 years | 18-45 years | 45-60 years | 60-80 years | ≥80 years |
|---|---|---|---|---|---|---|---|---|---|---|---|---|---|---|---|---|---|
| C1 | 0.00 | 0.00 | 0.00 | 0.00 | 0.00 | 0.00 | 0.00 | 0.00 | 0.00 | 0.00 | 0.00 | 0.00 | 0.00 | 0.00 | 0.00 | 0.00 | 0.00 |
| C2 | 0.00 | 0.00 | 0.00 | 0.00 | 0.00 | 0.00 | 0.00 | 0.00 | 0.00 | 0.00 | 0.00 | 0.00 | 0.00 | 0.00 | 0.00 | 0.00 | 0.00 |
| C3 | 0.00 | 0.00 | 0.00 | 0.00 | 0.00 | 0.00 | 0.00 | 0.00 | 0.00 | 0.00 | 0.00 | 0.00 | 0.00 | 0.00 | 0.00 | 0.00 | 0.00 |
| C4 | 0.00 | 0.00 | 0.00 | 0.00 | 0.00 | 0.00 | 0.00 | 0.00 | 0.00 | 0.00 | 0.00 | 0.00 | 0.00 | 0.00 | 0.00 | 0.00 | 0.00 |
| C5 | 0.00 | 0.00 | 0.00 | 0.00 | 0.00 | 0.00 | 0.00 | 0.00 | 0.00 | 0.00 | 0.00 | 0.00 | 0.00 | 0.00 | 0.00 | 0.00 | 0.00 |
| C6 | 0.00 | 0.00 | 0.00 | 0.00 | 0.00 | 0.00 | 0.00 | 0.00 | 0.00 | 0.00 | 0.00 | 0.00 | 0.00 | 0.00 | 0.00 | 0.00 | 0.00 |
| C7 | 0.00 | 0.00 | 0.00 | 0.00 | 0.00 | 0.00 | 0.00 | 0.00 | 0.00 | 0.00 | 0.00 | 0.00 | 0.00 | 0.00 | 0.00 | 0.00 | 0.00 |
| C8 | 0.00 | 0.00 | 0.00 | 0.00 | 0.00 | 0.00 | 0.00 | 0.00 | 0.00 | 0.00 | 0.00 | 0.00 | 0.00 | 0.00 | 0.00 | 0.00 | 0.00 |
| C9 | 0.00 | 0.00 | 0.00 | 0.00 | 0.00 | 0.00 | 0.00 | 0.00 | 0.00 | 0.00 | 0.00 | 0.00 | 0.00 | 0.00 | 0.00 | 0.00 | 0.00 |
| C10 | 0.00 | 0.00 | 0.00 | 0.00 | 0.00 | 0.00 | 0.00 | 0.00 | 0.00 | 0.00 | 0.00 | 0.00 | 0.00 | 0.00 | 0.00 | 0.00 | 0.00 |
| C11 | 0.00 | 0.00 | 0.00 | 0.00 | 0.00 | 0.00 | 0.00 | 0.00 | 0.00 | 0.00 | 0.00 | 0.00 | 0.00 | 0.00 | 0.00 | 0.00 | 0.00 |
| C12 | 0.00 | 0.00 | 0.00 | 0.00 | 0.00 | 0.00 | 0.00 | 0.00 | 0.00 | 0.00 | 0.00 | 0.00 | 0.00 | 0.00 | 0.00 | 0.00 | 0.00 |
| C13 | 0.00 | 0.00 | 0.00 | 0.00 | 0.00 | 0.00 | 0.00 | 0.00 | 0.00 | 0.00 | 0.00 | 0.00 | 0.00 | 0.00 | 0.00 | 0.00 | 0.00 |
| C14 | 0.00 | 0.00 | 0.00 | 0.00 | 0.00 | 0.00 | 0.00 | 0.00 | 0.00 | 0.00 | 0.00 | 0.00 | 0.00 | 0.00 | 0.00 | 0.00 | 0.00 |

| | | | | | | | | | | | | | | | | | |
|---|---|---|---|---|---|---|---|---|---|---|---|---|---|---|---|---|---|
| C15 | 0.00 | 0.00 | 0.00 | 0.00 | 0.00 | 0.00 | 0.00 | 0.00 | 0.00 | 0.00 | 0.00 | 0.00 | 0.00 | 0.00 | 0.00 | 0.00 | 0.00 |
| C16 | 0.00 | 0.00 | 0.00 | 0.00 | 0.00 | 0.00 | 0.00 | 0.00 | 0.00 | 0.00 | 0.00 | 0.00 | 0.00 | 0.00 | 0.00 | 0.00 | 0.00 |
| C17 | 0.00 | 0.00 | 0.00 | 0.00 | 0.00 | 0.00 | 0.00 | 0.00 | 0.00 | 0.00 | 0.00 | 0.00 | 0.00 | 0.00 | 0.00 | 0.00 | 0.00 |
| YXH | 0.00 | 0.00 | 0.00 | 0.00 | 0.00 | 0.00 | 0.00 | 0.00 | 0.00 | 0.00 | 0.00 | 0.00 | 0.00 | 0.00 | 0.00 | 0.00 | 0.00 |
| SQH | 0.00 | 0.00 | 0.00 | 0.00 | 0.00 | 0.00 | 0.00 | 0.00 | 0.00 | 0.00 | 0.00 | 0.00 | 0.00 | 0.00 | 0.00 | 0.00 | 0.00 |
| BSTH | 0.00 | 0.00 | 0.00 | 0.00 | 0.00 | 0.00 | 0.00 | 0.00 | 0.00 | 0.00 | 0.00 | 0.00 | 0.00 | 0.00 | 0.00 | 0.00 | 0.00 |
| ZH | 0.00 | 0.00 | 0.00 | 0.00 | 0.00 | 0.00 | 0.00 | 0.00 | 0.00 | 0.00 | 0.00 | 0.00 | 0.00 | 0.00 | 0.00 | 0.00 | 0.00 |
| PH | 0.00 | 0.00 | 0.00 | 0.00 | 0.00 | 0.00 | 0.00 | 0.00 | 0.00 | 0.00 | 0.00 | 0.00 | 0.00 | 0.00 | 0.00 | 0.00 | 0.00 |
| HBH | 0.00 | 0.00 | 0.00 | 0.00 | 0.00 | 0.00 | 0.00 | 0.00 | 0.00 | 0.00 | 0.00 | 0.00 | 0.00 | 0.00 | 0.00 | 0.00 | 0.00 |
| ZGH | 0.00 | 0.00 | 0.00 | 0.00 | 0.00 | 0.00 | 0.00 | 0.00 | 0.00 | 0.00 | 0.00 | 0.00 | 0.00 | 0.00 | 0.00 | 0.00 | 0.00 |
| TYH | 0.00 | 0.00 | 0.00 | 0.00 | 0.00 | 0.00 | 0.00 | 0.00 | 0.00 | 0.00 | 0.00 | 0.00 | 0.00 | 0.00 | 0.00 | 0.00 | 0.00 |
| SWLH | 0.00 | 0.00 | 0.00 | 0.00 | 0.00 | 0.00 | 0.00 | 0.00 | 0.00 | 0.00 | 0.00 | 0.00 | 0.00 | 0.00 | 0.00 | 0.00 | 0.00 |
| NFH | 0.00 | 0.00 | 0.00 | 0.00 | 0.00 | 0.00 | 0.00 | 0.00 | 0.00 | 0.00 | 0.00 | 0.00 | 0.00 | 0.00 | 0.00 | 0.00 | 0.00 |

## References


1. Fan, R.; Zhang, W.; Li, L.; Jia, L.; Zhao, J.; Zhao, Z.; Peng, S.; Yuan, X.; Chen, Y. Individual and synergistic toxic effects of carbendazim and chlorpyrifos on zebrafish embryonic development. *Chemosphere* **2021**, *280*, doi:10.1016/j.chemosphere.2021.130769.
2. Silva, A.R.R.; Cardoso, D.N.; Cruz, A.; Lourenço, J.; Mendo, S.; Soares, A.M.V.M.; Loureiro, S. Ecotoxicity and genotoxicity of a binary combination of triclosan and carbendazim to Daphnia magna. *Ecotoxicology and Environmental Safety* **2015**, *115*, 279-290, doi:10.1016/j.ecoenv.2015.02.022.
3. Ma, J.; Zheng, R.; Xu, L.; Wang, S. Differential Sensitivity of Two Green Algae, Scenedesmus obliqnus and Chlorella pyrenoidosa, to 12 Pesticides. *Ecotoxicology and Environmental Safety* **2002**, *52*, 57-61, doi:10.1006/eesa.2002.2146.
4. Richter, E.; Wick, A.; Ternes, T.A.; Coors, A. Ecotoxicity of climbazole, a fungicide contained in antidandruff shampoo. *Environmental Toxicology and Chemistry* **2013**, *32*, 2816-2825, doi:10.1002/etc.2367.
5. Li, M.-H. Comparative toxicities of 10 widely used biocides in three freshwater invertebrate species. *Chemistry and Ecology* **2019**, *35*, 472-482, doi:10.1080/02757540.2019.1579311.
6. Gustafson, A.L.; Stedman, D.B.; Ball, J.; Hillegass, J.M.; Flood, A.; Zhang, C.X.; Panzica-Kelly, J.; Cao, J.; Coburn, A.; Enright, B.P.; et al. Inter-laboratory assessment of a harmonized zebrafish developmental toxicology assay – Progress report on phase I. *Reproductive Toxicology* **2012**, *33*, 155-164, doi:10.1016/j.reprotox.2011.12.004.
7. Assress, H.A.; Nyoni, H.; Mamba, B.B.; Msagati, T.A.M. Occurrence and risk assessment of azole antifungal drugs in water and wastewater. *Ecotoxicology and Environmental Safety* **2020**, *187*, doi:10.1016/j.ecoenv.2019.109868.
8. Chen, Z.-F.; Ying, G.-G.; Jiang, Y.-X.; Yang, B.; Lai, H.-J.; Liu, Y.-S.; Pan, C.-G.; Peng, F.-Q. Photodegradation of the azole fungicide fluconazole in aqueous solution under UV-254: Kinetics, mechanistic investigations and toxicity evaluation. *Water Research* **2014**, *52*, 83-91, doi:10.1016/j.watres.2013.12.039.
9. Dobbins, L.L.; Usenko, S.; Brain, R.A.; Brooks, B.W. Probabilistic ecological hazard assessment of parabens using Daphnia magna and Pimephales promelas. *Environmental Toxicology and Chemistry* **2010**, *28*, 2744-2753, doi:10.1897/08-523.1.
10. Yamamoto, H.; Tamura, I.; Hirata, Y.; Kato, J.; Kagota, K.; Katsuki, S.; Yamamoto, A.; Kagami, Y.; Tatarazako, N. Aquatic toxicity and ecological risk assessment of seven parabens: Individual and additive approach. *Science of The Total Environment* **2011**, *410-411*, 102-111, doi:10.1016/j.scitotenv.2011.09.040.
11. PPDB. Carbendazim (Ref: BAS 346F). Available online:

https://sitem.herts.ac.uk/aeru/ppdb/en/Reports/116.htm (accessed on

12. Schönrath, I.; Schmidtkunz, C.; Küpper, K.; Weber, T.; Leng, G.; Kolossa-Gehring, M. Exposure of young German adults to the anti-dandruff agent climbazole from 2002 to 2022: Analysis of specific biomarkers in urinary samples. *Chemosphere* **2024**, *367*, doi:10.1016/j.chemosphere.2024.143611.
13. Evaluations of the Joint FAO/WHO Expert Committee on Food Additives(JECFA). Available online: https://apps.who.int/food-additives-contaminants-jecfa-database/Home/Chemical/3206 (accessed on
